# Supplementary material for: CryoEM structure of adenovirus type 3 fibre with desmoglein 2 shows an unusual mode of receptor engagement
Source: Nat Commun. 2019 Mar 12;10:1181. doi: 10.1038/s41467-019-09220-y (PMC6414520; doi:10.1038/s41467-019-09220-y)
Supplement: Supplementary file 1 — Supplementary Information [file 41467_2019_9220_MOESM1_ESM.pdf]

Cryo-EM structure of adenovirus type 3 fibre with desmoglein 2 shows an unsual mode of receptor engagement

E. Vassal-Stermann, G. Effantin *et al.*,

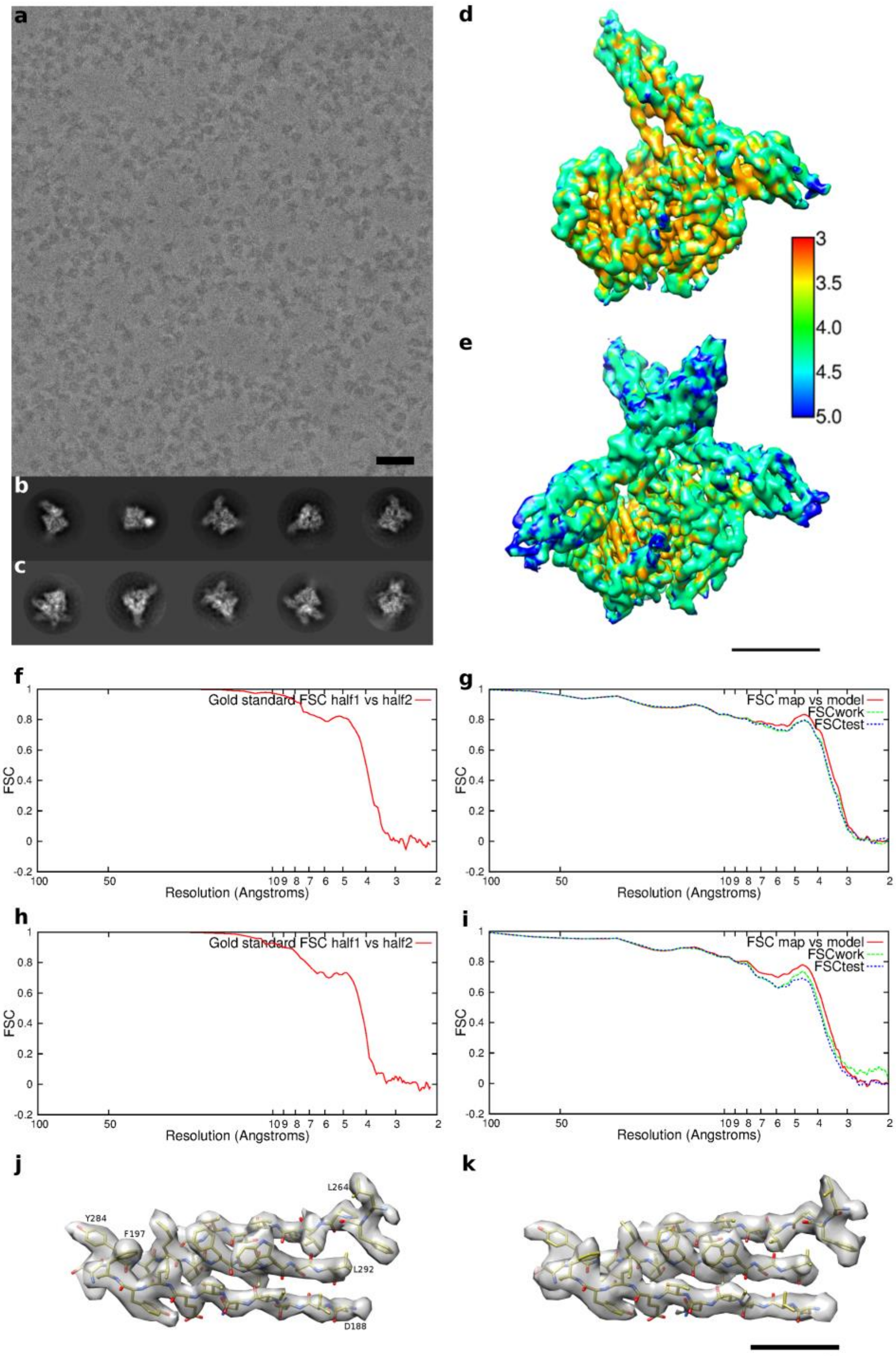

Supplementary Figure 1 : **a**- Electron micrograph of HAd3K/EC2-EC3 complexes. **b** & **c**- 2D class averages for HAd3K in complex with one (**b**) or two (**c**) EC2-EC3 modules. **d** & **e**- 3D maps of HAd3K in complex with one (**d**) or two (**e**) EC2-EC3 modules colored according to local resolution. **f** & **h**- Fourier shell correlation (FSC) curves of the final 3D reconstructions obtained for HAd3K in complex with either one (**f**) or two (**h**) EC2-EC3 modules, the resolutions using FSC = 0.143 are 3.5 and 3.8 Å respectively. **g** & **i**- Cross-validation FSC curves for the refined model versus the summed map (FSC map vs model), half map 1 (FSCwork) and 2 (FSCtest) for HAd3K in complex with one (**g**) or two (**i**) DSG2 modules. **j** & **k**- Electron density of beta sheets fitted with the refined structure for the 3D reconstructions of HAd3K in complex with one (**j**)or two (**k**) DSG2 modules. Scale bars are 200 (Panel **a**), 30 (Panels **d, e**) and 10 Å (Panels **j**, **k**).

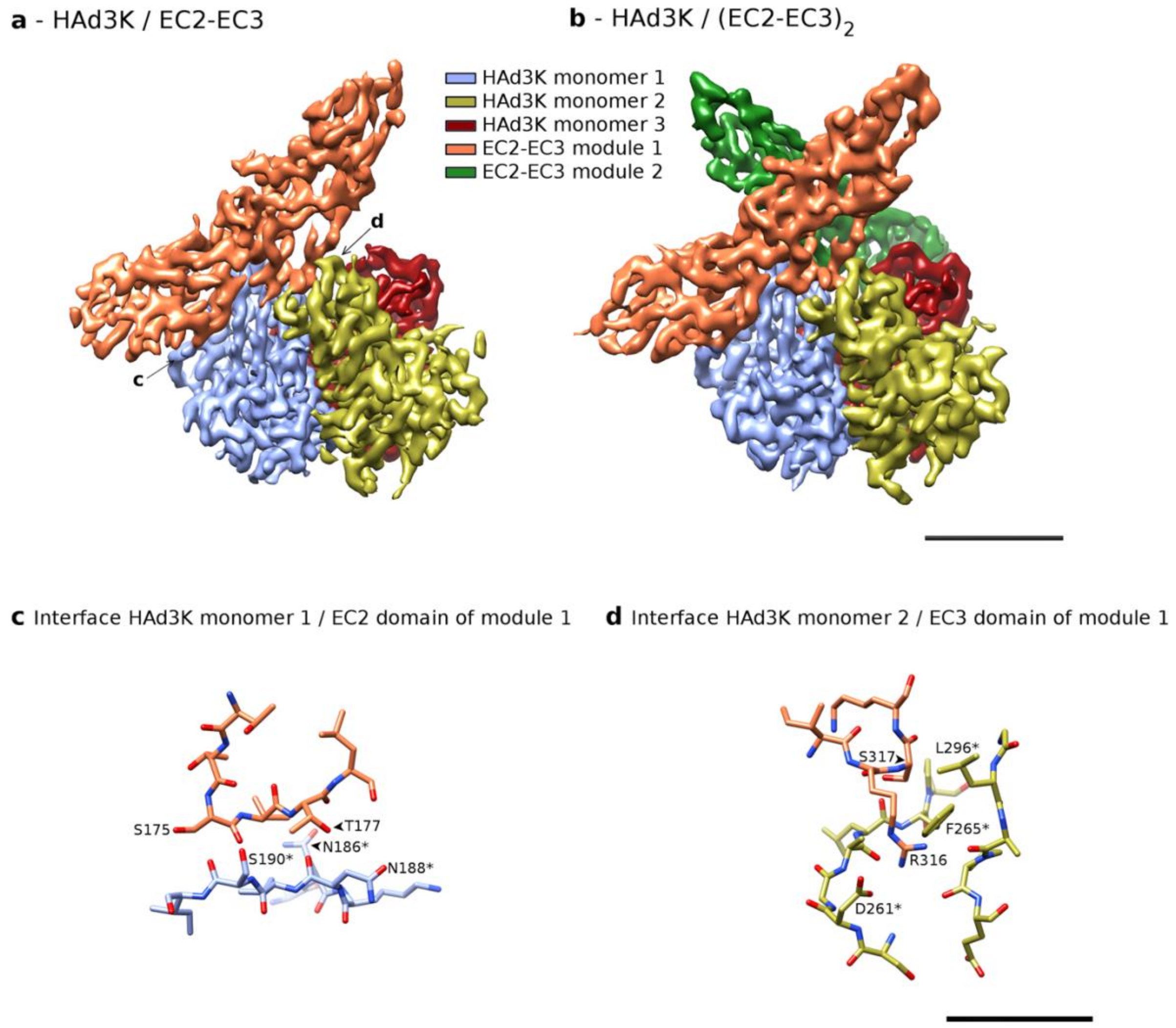

Supplementary Figure 2 : **a**, **b** - Isosurface views of the 3D reconstruction of HAd3K in complex with one (**a**) or two (**b**) EC2-EC3 modules. The subunits of both reconstructions are colored according to the color code. **c**, **d** – Close up views (arrows in (**a**)) for the 3D reconstruction of HAd3K in complex with one EC2-EC3 module. **c**- Zoom on residues at the interface of HAd3K monomer 1 and EC2 domain of EC2-EC3 module 1. **d**- Zoom on residues at the interface of HAd3K monomer 2 and EC3 domain of EC2-EC3 module1. Scale bars are 30 (Panels **a**, **b**) and 10 Å (Panels **c**, **d**). (*) Residue reported to reduce or abolish DSG2 binding in (Wang *et al.,* JVI 2013 : PMID 23946456)

**Cryo-EM data collection**		
Magnification	130k	
Pixel size (Å)	0.53	
Defocus Range (µm)	-0.5 – -1.0	
Voltage	300kV	
Exposure dose	~1.5 e^-/pixel/s	
Exposure time (s per frame)	0.175	
Number of frames	40	
Total dose (e^-/Å^2)	35	
**3D reconstruction summary**		
Box size (pixels)	200	
Micrographs	2855	
Autopicked particles	1799654	
Particles after 2D classification	646863	
	**HAd3K with one DSG2 module**	**HAd3K with two DSG2 modules**
Particles included in the final 3D map	139958	78925
Resolution (Å) at FSC = 0.143	3.5	3.8
**Model refinement statistics**		
Resolution (Å) between atomic model and cryo-EM map	3.6 (FSC=0.5)	3.8 (FSC=0.5)
Ramachandran favored	92.0 %	90.0 %
Ramachandran outliers	0.0 %	0.0 %
Rotamer outliers	0.0 %	0.0 %
C-beta deviations	0	0
Clashscore	3.0	4.0
RMS(bonds)	0.0050	0.0051
RMS(angles)	1.17	1.20
MolProbity score	1.57	1.71

Supplementary Table 1: Data collection and refinement statistics

Residue Number	HAd3k/Ec2-Ec3	HAd3k/(Ec2-Ec3)$_2$ Module 1	HAd3k/(Ec2-Ec3)$_2$ Module 2
Ec2 LEU 122	✓	✓	✓
Ec2 ALA 124	✓	X	✓
Ec2 ALA 125	✓	✓	✓
Ec2 HIS 126	✓	✓	✓
Ec2 PRO 159	✓	✓	✓
Ec2 PRO 160	✓	✓	✓
Ec2 TYR 163	✓	✓	✓
Ec2 ASN 165	X	✓	✓
Ec2 LYS 166	X	✓	✓
Ec2 TYR 172	✓	✓	✓
Ec2 THR 173	✓	✓	X
Ec2 THR 174	✓H	✓H	✓
Ec2 SER 175	✓H	✓H	✓H
Ec2 VAL 176	✓	✓	✓
Ec2 THR 177	✓H	✓H	✓H
Ec2 LEU 178	✓	✓	✓
Ec2 ASP 179	✓	✓	✓S
Ec2 GLU 182	✓	X	✓
Ec3 SER 314	✓	X	✓
Ec3 ILE 315	✓	X	X
Ec3 LYS 318	✓	X	✓
HAd3k ILE 145	✓	✓	✓
HAd3k GLU 146	X	X	✓
HAd3k TYR 147	✓	✓	✓
HAd3k GLY 148	✓	✓	✓
HAd3k GLN 150	X	✓	✓
HAd3k TYR 179	✓H	✓H	✓
HAd3k LEU 183	✓	✓	✓
HAd3k ASN 186 *	✓	✓	✓
HAd3k LYS 187	✓	✓	✓S
HAd3k ASN 188	✓H	✓H	✓H
HAd3k VAL 189 *	✓	✓	✓
HAd3k SER 190 *	✓H	✓H	✓H
HAd3k ILE 191	✓	✓	✓
HAd3k ASN 192	✓	✓	✓
HAd3k GLU 268	X	X	✓
HAd3k LEU 289	✓	X	✓
HAd3k ASN 293	✓	✓	✓

Residues involved in the first interface of HAd3k with DSG2 identified by the PISA software (Table 2, first page)

Residue Number	Had3k/Ec2-Ec3	Had3k/(Ec2-Ec3)$_2$ Module 1	Had3k/(Ec2-Ec3)$_2$ Module 2
Ec3 ALA 306	X	✓	✓
Ec3 PHE 311	✓	✓	✓
Ec3 HIS 312	X	✓	✓
Ec3 LYS 313	✓	✓	✓
Ec3 ARG 316	✓H/S	✓H/S	✓H/S
Ec3 SER 317	✓H	✓H	✓H
Ec3 TYR 319	✓	✓	✓
Ec3 LYS 320	✓	✓	✓H
Ec3 PRO 321	✓	✓	✓
HAd3k LYS 187	✓	✓	✓
HAd3k ALA 259	✓	✓	✓
HAd3k ASP 261 *	✓H/S	✓H/S	✓H/S
HAd3k GLY 262	✓	✓	✓
HAd3k ALA 263	✓	✓	✓H
HAd3k LEU 264	✓H	✓H	✓H
HAd3k PHE 265 *	✓	✓	✓
HAd3k PRO 266	✓	✓	✓
HAd3k ASN 293	✓	✓	✓
HAd3k ALA 294	✓	✓	✓
HAd3k GLY 295	✓	✓	✓
HAd3k LEU 296 *	✓	✓	✓
HAd3k ALA 297	✓H	✓	✓
HAd3k PRO 298	✓	✓	✓H
HAd3k GLU 299	X	X	✓

Residues involved in the second interface of HAd3k with DSG2 identified by the PISA software (Table2, second page)

✓ Residue found at the interface
X Residue not found at the interface

H Residue found to form a putative hydrogen bound

S Residue found to form a putative salt bridge

* Residue reported to reduce or abolish DSG2 binding in (Wang *et al.,* JVI 2013 : PMID 23946456)

Supplementary Table 2: Interfacing residues between HAd3K and EC2 or EC3 domain of DSG2 identified by the PISA software

Primer Name	Primer Sequence
Ad3K-FWD-BamHI	TACATCGGATCCAGCTAATTCTATTGCACTTAAGAATAA
Ad3K-RVS-NotI	TACATCGCGGCCGCTCAGTCATCTTCTCTAATATAGGAAAAG
MBP- FWD-ATG-NdeI	TAGATCCATATGAAAATCGAAGAAGGTAAACTGG
EC3-RVS-KpnI	TAGATCGGTACCCTAAATGCCTTCTTTCACATTTTTCAC

Supplementary Table 3: Primers used for cloning in pET-Duet
